# Pros and Cons of Alpha versus Beta Bone Seeking Agents in the Treatment of Cancer Pain

**DOI:** 10.1055/s-0043-1774731

**Published:** 2023-12-04

**Authors:** Knut Liepe

**Affiliations:** 1 Department of Nuclear Medicine, Klinikum Frankfurt (Oder), Brandenburg, Germany

Skeletal metastases occur in many types of solid malignant tumors, especially in advanced stage of prostate, breast, and lung cancers. The resulting bone pain affects patient's quality of life and requires effective treatment. Only osteoblastic bone metastases are suitable for treatment with bone-seeking agents. Typical tumors are prostate cancer with 65 to 85% of bone metastases, breast cancer with 65 to 75%, and small cell lung cancer with 34 to 50%, respectively. 1 The mechanisms involved in bone pain are poorly understood, 2 but are likely to be a consequence of osteolysis (bone breakdown). 3 Infiltration of the bone trabeculae and matrix by tumor osteolysis is one of the physical factors. Pain may result from instability-based microfractures and stretching of the periosteum by tumor growth. 4 The pathophysiological mechanisms of pain include stimulation of free nerve endings in the endosteum by a variety of chemical mediators like bradykinin, prostaglandin, histamine, interleukin, and tumor necrosis factor. 4 5

Currently, the majority of researchers prefer α emitters, which are highly effective, requiring one to four deoxyribonucleic acid (DNA) hits to evoke cell death, compared with β emitters, which require greater than 1,000 DNA hits. 6 Alphas have the advantage of high linear energy transfer and a short range, enabling moderate bone marrow toxicity. Alphas have the disadvantage of a short range from 40 to 100 μm. Therefore alphas can interact only with four to six cell lines. 7 In therapies such as prostate-specific membrane antigen (PSMA) or DOTATOC ([DOTA(0)-Phe(1)-Tyr(3)]octreotide) that guide α emitters directly to tumor cells, the short range can be discussed as an advantage. 8 In therapies like bone pain palliation or microsphere therapy, radionuclides are deposited in the tissue surrounding the tumor. Here, the short range of α emitters is considered a disadvantage and a longer range of β emitters might lead to higher doses of tumor absorbed. A long range leads to higher rates of crossfire and affects cells in greater distance from the source. It can be supposed that the higher the energy of β radiation is, the higher is the bone marrow toxicity. Interestingly, clinical data show this neither in a direct comparison of ^188^ Re-HEDP (E _max_ 2.12 MeV) and ^153^ Sm-EDTMP (E _max_ 0.81 MeV) 9 nor in dose calculation. 10 In future, more dosimetric data of radiation-absorbed doses to tumor and surrounding tissue are needed to compare therapeutically relevant α and β emitters.

A possible reason for theoretical and in vivo differences is the bystander effect. Blyth and Sykes defined bystander response as “radiation-induced, signal-mediated effects in un-irradiated cells within an irradiated volume.” 11 These signal mediated bystander effects include cell death, DNA damage, chromatid aberrations, genomic instability, transformation, differentiation, proliferation, gene expression, cell cycle, invasion, and radio-adaptive responses. 12 The primary mechanisms involved are release of signaling molecules 13 and direct intercellular communication via gap junctions. 14 Cell proximity has also been shown to be necessary for proliferative bystander responses. 15 Reactive oxygen species, reactive nitrogen species, calcium, and cytokines have been implicated in bystander signaling. 16 Cell proximity is necessary for proliferative bystander responses. 15

Overall survival is the most important outcome for patients and clinicians. The well-designed randomized Alpharadin in Symptomatic Prostate Cancer Patients (ALSYMPCA) trial showed a survival benefit of 3.6 months for the α emitter ^223^ Ra. 17 Trials aiming in determination of overall survival are limited for β emitters. Biersack et al 18 showed in a small study a prolongation of overall survival from 4.5 to 15.7 months after three cycles of ^188^ Re-HEDP compared with a single treatment.

An interesting therapeutic strategy may be the combination of α and β emitters or the combination of tumor- and bone-guided tracers, for example, ^223^ Ra and ^177^ Lu-PSMA. 19 For nuclear medicine to become a key player in pain palliation and systemic tumor therapy, we need well-designed trials like ALSYMPCA 20 or VISION 21 aimed at determining the overall survival and comparison studies like TheraP 22 to establish our methods.
